# Widely targeted metabolomics reveals the effect of different raw materials and drying methods on the quality of instant tea

**DOI:** 10.3389/fnut.2023.1236216

**Published:** 2023-10-11

**Authors:** Jian-Chang Jin, Shuang Liang, Shang-Xiong Qi, Ping Tang, Jian-Xin Chen, Quan-Sheng Chen, Yan-Feng Chen, Jun-Feng Yin, Yong-Quan Xu

**Affiliations:** ^1^College of Biological and Environmental Engineering, Key Laboratory of Pollution Exposure and Health Intervention of Zhejiang Province, Zhejiang Shuren University, Hangzhou, China; ^2^Key Laboratory of Biology, Genetics and Breeding of Special Economic Animals and Plants, National Engineering Research Center for Tea Processing, Tea Research Institute Chinese Academy of Agricultural Sciences, Ministry of Agriculture and Rural Affairs, Hangzhou, China; ^3^Eastsign Foods (Quzhou) Co., Ltd., Quzhou, China; ^4^Hangzhou Vocational and Technical College, Hangzhou, China; ^5^College of Ocean Food and Biological Engineering, Jimei University, Xiamen, China; ^6^Unibioche Food Tech Co., Ltd., Hangzhou, China

**Keywords:** instant tea, raw tea materials, drying, flavonoids, aroma

## Abstract

**Introduction:**

Instant teas are particularly rich in tea polyphenols and caffeine and have great potential as food ingredients or additives to improve the quality of food and enhance their nutritional and commercial value.

**Methods:**

To determine the relationships between raw material, drying method, and sensory and other quality attributes, instant teas were prepared from three tea varieties, namely black, green and jasmine tea, using two drying methods, namely spray-drying (SD) and freeze-drying (FD).

**Results:**

Both the raw tea material and drying method influenced the quality of the finished instant teas. Black tea was quality stable under two drying, while green tea taste deteriorated much after SD. Jasmine tea must be produced from FD due to huge aroma deterioration after SD. FD produced instant tea with higher sensory quality, which was attributed to the lower processing temperature. Chemical compositional analysis and widely targeted metabolomics revealed that SD caused greater degradation of tea biochemical components. The flavonoids content changed markedly after drying, and metabolomics, combined with OPLS-DA, was able to differentiate the three varieties of tea. Instant tea preparations via SD often lost a large proportion of the original tea aroma compounds, but FD minimized the loss of floral and fruity aroma compounds. Changes in the tea flavonoids composition, especially during drying, contributed to the flavor development of instant tea.

**Discussion:**

These results will provide an practicle method for high-quality instant tea production through choosing proper raw tea material and lowering down drying temperature with non-thermal technologies like FD.

## 1. Introduction

Tea has long been recognized for its unique color, flavor and health properties, and has been described as the most pleasant and popular of beverages (1). In 2022, global tea production was 6.397 million tons, a decrease of 54,000 tons compared with 2021. Traditionally, a time-consuming and complex process is needed to offer consumers a fragrant tea with sweet and mellow taste, which contains both rigorous water preparation paying attention to water quality and temperature and strict tea brewing paying attention to tea-to-water ratio and brewing time (2). To simplify tea preparation process, instant tea with characteristic flavor and satisfying solubility come into market. Instant tea is a powdered form of tea infusion, manufactured from tea leaf extracts of the six tea varieties (black, green, oolong, etc.). In addition to drinking, instant tea can also be added to other foods to improve flavor, increase nutritional quality and extend shelf-life, with great potential for new applications (3).

The sensory quality and health benefits of tea are clearly related to the inherent primary and secondary plant metabolites, such as polyphenols, amino acids, alkaloids, tea polysaccharides and tea saponins (4). Tea polyphenols, the general term for phenolic compounds in tea, are the main bioactive compounds in tea and usually comprise 18–36% of tea leaf dry weight. Due to remarkable health benefits and specific structural properties, tea polyphenols are widely used in the food industry (5). Tea flavonoids, the most studied functional phenolic components, are the important contributor to the taste and multiple health-promoting effects, particularly anti-inflammatory, antioxidant, antimicrobial, neuroprotective, antihypertensive, and anti-carcinogenic properties (6). As the most abundant secondary metabolites, tea flavonoids comprise a large group of structurally diverse compounds with a variety of basic skeleton (6), namely flavan-3-ols (such as catechins, theaflavins, thearubigins, and proanthocyanidins), flavonols (such as quercetin, kampferol, myricetin, and their glycosides) and flavones (such as luteolin and apigenin). Tea flavonoids have attracted much attention in recent years because of their major contribution as complementary and alternative medicines, and tea would be the rich source of flavonoids for daily intake (7).

Past studies on instant tea focused on the mechanisms that influence quality and sensory attributes in each procedures. During the extraction procedure, choosing processed tea (as opposed to green) as the raw material would compromise the quality of the instant tea, because the chemical components in processed tea have been degraded and modified by heat-treatment (8). One way of reducing flavor components degradation is to use fermented dhool (particles resulting from rolling of green tea leaves) as the raw material (8). Filtration and concentration of the extract resulted in decreased concentrations of geraniol, indole, *β*-damascenone, methyl jasmonate, *trans*-nerolidol, and safranal (9). Ultrafiltration of the extract before drying promoted the loss of aroma volatiles, by removing some of the tea polyphenols, which would otherwise combine with and help retain the volatiles (9).

Drying is the key step of instant tea production. The drying technology of instant tea after water extraction is well-established, usually based on three drying methods: spray-drying (SD), freeze-drying (FD), or vacuum-drying. SD uses the high temperature/very short time approach for rapid evaporation of water, which has advantages of satisfying powder characteristics, continuous production and large throughput (10). However, when processing tea infusion into instant tea powder, high temperatures involved in SD can alter the content and composition of aroma components by evaporation, oxidation and thermal degradation (8, 11, 12). By contrast, FD involves removal of water by sublimation of ice under a vacuum, at temperatures below the freezing point of water (13, 14). This low temperature minimizes thermal degradation and evaporation of flavor components (15). Although FD effectively minimizes loss of flavor components, it requires costly freezing and vacuum systems, which have much higher energy consumption and capital costs than SD.

Since high temperature is the main cause of aroma compound loss and quality deterioration, reducing the processing temperature is considered a promising way to maximize the quality of instant tea (1). Recently, electrostatic spray drying, which operates at a much lower temperature (outlet air temperature 38–42°C) than conventional SD, was shown to be comparable to FD in terms of sensory quality and productivity, although most aroma compounds were less abundant than after FD (12). In this study, three normal raw tea materials, black tea (BT), green tea (GT) and jasmine tea (JT; GT scented with jasmine flowers so the tea absorbs the jasmine scent), were subjected to analysis of the relationships between sensory quality and quality attributes when processed into instant teas (IBT, IGT, and IJT) by SD or FD. The influence of the different raw materials and drying methods on the organoleptic properties, chemical composition and physicochemical properties of instant tea was determined. Metabolomics analysis was applied to identify differential metabolites between drying methods and elucidate the relationships and interactions between flavonoids and volatile compounds. The findings will provide a theoretical and scientific basis for improved quality control of instant tea during the drying procedure.

## 2. Materials and methods

### 2.1. Reagents and materials

Black tea, green tea and jasmine tea were from Eastsign Foods (Quzhou) Co., Ltd. (Quzhou, China). Purified water was from Hangzhou Wahaha Beverage Co., Ltd. (Hangzhou, Zhejiang, China), which had a total dissolved solids of 1.768 mg/L and a pH of 6.74.

Gallic acid (GA), gallocatechin (GC), epigallocatechin (EGC), catechin (C), epicatechin (EC), gallocatechin gallate (GCG), epigallocatechin gallate (EGCG), catechin gallate (CG), epicatechin gallate (ECG), theaflavin (TF), theaflavin-3-gallate (TF-3-G), theaflavin-3-gallate (TF-3-G) and theaflavin-3,3-digallate (TFDG) were from Shanghai Yuanye Biotechnology Co., Ltd. (China) with purity ≥95%. *N*-Alkanes for linear retention index (RI) determination and internal standard solution (decanoic acid ethyl ester) were from TCI (Shanghai) Development Co., Ltd. (Shanghai, China). Ninhydrin, stannous chloride, sodium carbonate, disodium hydrogen phosphate, potassium dihydrogen phosphate and disodium EDTA were all analytical grade from local suppliers. Acetic acid and acetonitrile were chromatographic grade, from local suppliers.

### 2.2. Preparation of instant tea

Process parameters was set as follows. Tea-to-water ratio was 1:10, extraction temperature for BT was 90°C, and for GT and JT was 75–80°C. Tea leaves (5 kg) were put in an extraction tank (diameter of 30 cm, height of 1.2 m) and were extracted with hot water, impregnated for 30 min, then extraction started to slowly extract the impregnated. The total soaking time was 3–4 h. The resulting extract concentrations were 6% (w/v) for BT, 8% (w/v) for GT and 10% (w/v) for JT. After centrifugation, the three tea extracts were used to prepare tea powders through SD and FD. For SD, the 50-type spray drying tower (D-300, from Changsheng Dryer Manufacturing Co., Ltd., Wuxi, China) had an inlet air temperature of 170°C and an outlet air temperature of 100°C. For FD, the vacuum freeze dryer (ZG-200, from Hangznou Innovative Vacuum Freeze-drying Plant, Hangzhou, China) was set to the following parameters (Table 1), which was from a mature large-scale FD process:

**Table 1:** Parameters of the vacuum freeze dryer for large-scale freeze-drying (FD) process.

Stage	Accumulated drying time/min	Operation time/min	Temperature (partition)/°C	Vacuum degree/Pa
1	30	30	20	70–100
2	90	60	30	
3	270	180	30	
4	300	30	40	
5	1,080	780	40	
6	1,110	30	50	
7	1830	720	50	

### 2.3. Sensory evaluation

Tea solution preparation were referred to “5.3.2.7 Powdered tea (Cylindrical cup evaluation method)” in Chinese standard GB/T 23776-2018 Methodology for sensory evaluation of tea with slight modification. The three varieties of instant tea powder (0.5 g) were each mixed into 100 mL of pure water at 50°C, and stayed for 1 min. To determine the sensory differences among the instant tea, evaluation of the appearance, liquor color, aroma and taste was scored on a 100-point scale, performed by five fully trained panelists (three men and two women, 26–52 years old) with certificates (four senior and one intermediate tea assessor) for tea quality evaluation from the Tea Scientific Society of China. There were three replicate ratings for each treatment.

### 2.4. Chemical and physical analysis of raw materials and instant teas

The test samples were prepared according to the method “3.4.3.2 Preparation of Test Solution” in Chinese standard GB/T 8313-2018. Determination of tea polyphenols was by the Folin phenol method according to Chinese standard GB/T 8313-2018. Total amino acids were determined by the ninhydrin colorimetric method according to Chinese standard GB/T 8314-2013. Determination of theaflavins (TFs) was by high-performance liquid chromatography (HPLC) according to Chinese standard GB/T 30483-2013.

Catechins, caffeine, and gallic acid were determined by HPLC (LC-20D, Shimadzu Kyoto, Japan) (16), fitted with a Symmetry C18 column (5 μm, 4.6 mm × 250 mm; Waters, Milford, MA). The mobile phases were 2% (v/v) aqueous acetic acid (A) and acetonitrile (B). The linear elution gradient was: 0–3 min, 6.5%B; 19 min, 15%B; 25 min, 25%B; 30–35 min, 6.5%B, at a flow rate of 1 mL/min, a column temperature of 40°C, UV detection at 280 nm and injection volume 10 μL. Samples were filtered before injection using 0.45 μm microporous membrane filters.

Color differences (CIELAB L*, a* and b* values) were measured by a CM-5 color difference meter (Konica Minolta, Tokyo, Japan). L* represents brightness, a* represents the red/green balance (+*a* is red, −*a* is green). b* represents the yellow/blue balance (+*b* is yellow, −*b* is blue).

### 2.5. Extraction of volatiles using headspace-solid phase microextraction (HS-SPME)

The extraction of volatile compounds from green tea was performed using HS-SPME (50/30 μm divinylbenzene/carboxen/polydimethylsiloxane; CAR/PDMS/DVB) fiber (catalog No. 57328-U; Supelco, Bellefonte, PA) (17). Tea sample (0.5 g), boiling water (5 mL), and the internal standard, decanoic acid ethyl ester (10 mg/L, 10 μL), were added to 20 mL glass headspace vials by injection. The samples were kept in a water bath at 60°C to equilibrate for 5 min, then extracted with the SPME fiber for 60 min. After sampling of the headspace volatiles, the SPME fiber was inserted into the GC injector in splitless mode, and held there for 5 min to allow analyte thermal desorption at 250°C.

### 2.6. GC–MS analysis

According to our previous study (17), GC–MS analysis were performed on a 7890B GC, coupled with an HP-5 ms Ultra Inert capillary column (30 m × 0.25 mm × 0.25 μm) and a 7000C MS (Agilent Technologies, Santa Clara, CA). The GC analysis conditions were: Carrier gas, high-purity helium at a flow rate of 1 mL/min; injection port temperature, 250°C; temperature program, initial column temperature 50°C, held for 5 min, increased to 150°C at 5°C/min, held for 2 min, increased to 270°C at 10°C/min, maintained for 6 min; No shunting. MS analysis conditions: electron impact mode; electron energy 70 eV; ion source temperature 230°C; transmission line temperature 270°C; and quality scanning range 35–400 amu.

The volatile compounds were identified by comparing retention indices (RIs) with those of authentic standards, or mass spectra with reference spectra in the Wiley and NIST14 libraries. The RIs were determined by comparison with a homologous series of alkanes (C_5_-C_30_) and identification was achieved by comparing the mass spectra. The relative quantification of volatile compounds with internal standard (IS) was calculated using the following equation: volatile compound concentration = (volatile compound peak area/IS peak area) × IS concentration. To compare the contribution of individual volatiles to the overall aroma profile, the relative odor activity value (rOAV) of each volatile was calculated by dividing its concentration by its olfactory threshold in water, obtained from the literature (18).

### 2.7. Dry sample extraction for UPLC–MS/MS analysis

The freeze-dried tea samples were ground into powder (30 Hz, 1.5 min), then powdered sample (50 mg) was extracted with pre-cooled 70% methanol/water (1,200 μL; −20°C), vortexing for 30 s, every 30 min, repeated five times. After centrifugation at 15984 *g* (or 12,000 rpm with a centrifugal radius of 10 cm) for 3 min, the supernatant was filtered using a microporous filter membrane (0.22 μm) then stored until needed for UPLC–MS/MS analysis.

### 2.8. UPLC–MS/MS analysis

The sample extracts were analyzed using an UPLC–MS/MS system (ExionLC AD, Sciex, Toronto, Canada).

The UPLC was fitted with an Agilent SB-C18 column (1.8 μm, 2.1 mm × 100 mm). The mobile phases were ultrapure water/0.1% formic acid (A) and acetonitrile/0.1% formic acid (B). The elution gradient was: Initially 5% B, then increased linearly to 95% B at 9 min, held for 1 min, then decreased linearly to 5% B at 11.10 min, held until 14 min. The flow rate was 0.35 mL/min, column temperature 40°C and injection volume 2 μL.

The MS/MS system had an electrospray (ESI) turbo interface (temperature 500°C; ion spray voltage 5,500 V for positive ion mode; −4,500 V for negative ion mode). The ion source gas I, gas II, and curtain gas were set to 50, 60, and 25 psi respectively, and the collision induced ionization parameters were set to high. QQQ scanning used MRM mode with the collision gas (nitrogen) set to medium. Optimization of the clustering potential (DP) and collision energy (CE) enabled determination of the DP and CE of each MRM ion pair. A specific set of MRM ion pairs was monitored, based on the metabolite eluting at any given time.

Identification of metabolites was carried out by comparing their MS2 spectra with a tea database developed in our laboratory (MWDB) and with the human metabolome database.^1^ Isotopic signals, repetitive signals containing K^+^ ions, Na^+^ ions, and NH_4_^+^ ions, as well as repetitive signals of fragment ions of higher molecular weight substances were removed. Then, the data were normalized to the sample weight, before carrying out statistical analysis.

### 2.9. Statistical analysis

Three replicates of all samples were analyzed. Data are presented as the mean ± standard deviation and analysis of variance was performed using SPSS 25.0 software. Comparison of means between samples was performed using the Least Significant Difference (LSD) method. The biochemical compositional map of each treatment was plotted using GraphPad Prism 8 (GraphPad Software, San Diego, CA). Principal components analysis (PCA) and supervised orthogonal partial least squares-discriminant analysis (OPLS-DA) were performed using SIMCA 14.1 software (Umetrics, Umeå, Sweden). Heatmaps were produced by Multi Experiment Viewer (version 4.7.4) software.

## 3. Results and discussion

### 3.1. Effect of drying method on the sensory quality of instant teas

The sensory evaluation and solubility comparison results of instant tea powder prepared by SD and FD are shown in Table 2. FD tea powder was crystalline, whereas SD tea powder was amorphous. The solubility of FD tea powder was higher than that of SD tea powder, indicating that the two drying methods have different influence on the solid structure of tea powder. Drying with SD turned black tea powder brown and jasmine tea powder gray, indicating the existence of heat-induced damage to the tea components. The overall sensory score of SD green tea powder was slightly lower than that of FD green tea powder due to lower solubility of the former, however, the two drying methods had little effect on the color of green tea powder. Increasing the inlet temperature of SD can decrease the solubility of the resulting tea powder (19). Increasing the temperature from 145°C to 165°C increased the a* value by 40% and decreased the L* value by 5% (19), i.e., the tea powder darkened at higher inlet temperatures. Therefore, to minimize color changes in tea powder, the SD inlet temperature in drying procedure should be reduced while ensuring spray efficiency.

**Table 2:** Sensory evaluation results of instant tea with different drying methods.

Tea varieties^*^	Instant tea powder			Instant tea infusion					
	Appearance	Solubility	Score	Liquor color	Score	Aroma	Score	Taste	Score
FD-Black tea	Red, uniform, crystalline	High	84	Red, little bright	87	Sweet, little high	88	Mellow, little sweet	88
SD-Black tea	Reddish brown, uniform, powdery	Low	82	Little red, little bright, clear	82	Little sweet	83	Less mellow, less sweet	84
FD-Green tea	Yellow green, uniform, crystalline	High	85	Green yellow, little bright	84	Little characteristic tea aroma	85	Less mellow, with steamed tea flavor	84
SD-Green tea	Yellow green, uniform, powdery	Low	84	Green yellow, little bright	85	Less characteristic tea aroma	81	Little coarse and astringent, bitter	79
FD-Jasmine tea	Yellow, uniform, crystalline	High	85	Yellow, little bright	88	Flowery, rich	89	Less mellow	87
SD-Jasmine tea	Yellow with gray, uniform, powdery	Low	83	Yellow, less bright	87	With flowery note	83	Less mellow, with astringent taste	82

^*^Tea varieties are the instant teas prepared from three tea varieties using two drying methods, namely spray-drying (SD) and freeze-drying (FD).

The instant tea via two drying methods was observed totally different after redissolution in water. As for liquor color, FD tea solution remained normal red color, whereas SD tea solution was reddish-brown. In addition, the aroma of all three FD tea infusions was better than the SD infusions. As for aroma, SD tea had the same aromas as the corresponding FD, but with lower intensity. Similarly, the taste quality of the FD tea was better than that of the SD as for taste. SD green tea was more bitter, with the lowest score, and SD black and jasmine tea were more astringent. FD retained more flavor and bioactive compounds in instant green tea (20), whereas that of SD tea was much weaker (21). Overall, SD produced instant tea with a browner color, lower solubility, weaker aroma and a stronger bitter taste. FD instant tea maintained the flavor quality of the teas and all sensory, thus were better than SD ones. Black tea was more stable in quality changes between two drying methods. Green tea liquor color can be well maintained while its taste deteriorates much after SD. Jasmine tea liquor color can also be well maintained, while its aroma deteriorates much more after SD. Therefore, jasmine tea must be produced from FD to maintain its characteristic aroma.

The two drying methods had significant effect on the brightness (L*), red-green balance (a*), and yellow-blue balance (b*) of the instant tea solutions (Supplementary Figure S1). SD significantly increased L* for IBT solution, but significantly decreased a* and b* (Supplementary Figures S1A–C), i.e., SD reduced the redness and yellowness of tea solution and decreased its quality, which is in agreement with the sensory evaluation results. SD significantly increased L* for IJT solution, same as IBT and IGT. However, a* and b* increased significantly, i.e., the redness and yellowness of IJT solution increased, unlike IBT and IGT. SD significantly increased the L* of IGT and significantly decreased a* and b*, reducing the redness and yellowness (Supplementary Figures S1A–C), but increasing the greenness, which is in agreement with the sensory evaluation results. Overall, SD reduced the color quality of IBT and IJT, but maintained the color quality of IGT solution.

### 3.2. Effect of drying method on the chemical components related to the quality of instant teas

**Figure 1:**
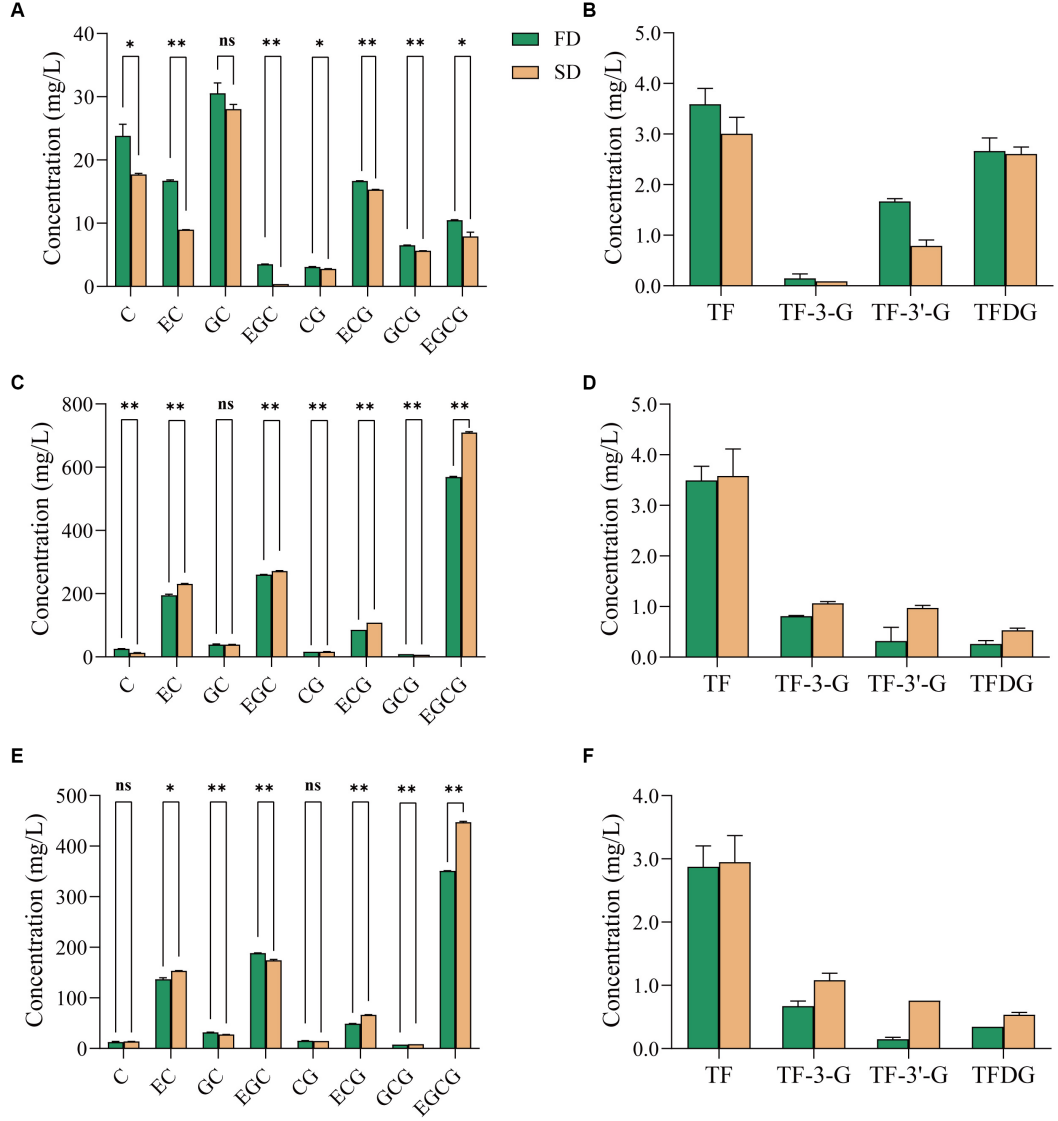
Effect of different drying methods on the content of catechins and theaflavins in instant tea powder. **(A,B)** Black tea. **(C,D)** Green tea. **(E,F)** Jasmine tea. FD, freeze drying; SD, spray drying.

SD and FD had significantly different effects on the content of tea polyphenols, amino acids, and caffeine in the three instant teas (Figure 1). The contents of tea polyphenols, amino acids and caffeine in IBT after SD were significantly lower than that after FD. SD significantly reduced the amino acids of IGT (Supplementary Figure S1D), but significantly increased the tea polyphenol and caffeine contents (Supplementary Figures S1E,F), which was consistent with the stronger bitterness and astringency of SD-GT. SD significantly decreased the content of amino acids in IJT, but significantly increased the contents of tea polyphenols and caffeine, same as IGT. Therefore, SD significantly reduced the contents of the main quality-related chemical components of IBT, compared with FD. Although SD increased the contents of tea polyphenols and caffeine in IGT and IJT, it also consequently strengthened their undesirable bitter taste.

SD and FD had significant but slightly different effect on the catechins composition of the three instant teas. SD had no significant effect on the GC content of IBT (Figure 1A), but reduced those of the other seven catechins compared with FD. For IGT (Figure 1C), the concentrations of C and GCG under FD were significantly higher than those after SD, but the concentrations of EC, EGC, CG, ECG and EGCG increased significantly after SD. The increased content of five catechins is consistent with the increased content of tea polyphenols and indicates that limited catechins oxidation occurred during SD. Neither treatment had significant effect on the GC content. Neither treatment had a significant effect on the C and CG contents of IJT (Figure 1E), whereas those of GC and EGC were higher after FD and those of EC, EC, GGC and EGCG were higher after SD. The increased astringency of IGT and IJT after SD is consistent with the increased contents of the astringent galloyl ester catechins (ECG and EGCG). The above analysis indicates that the composition changes under thermal production is complicated, but from sensory scores FD is more preferable for instant tea production.

To further analyze the effect of the temperature differences between FD and SD on the quality of instant tea powder, the generation of TFs was compared between the two treatments (Figures 1B,D,F). The concentration of four TFs in IBT after SD was lower than after FD (Figure 1B). TFs are sensitive to high temperature, and their concentration decreases with increased extraction, concentration and drying temperatures (22). In this study, since the SD temperature was much higher than that of FD, the former resulted in a greater loss of TFs. TFs appear to be further oxidized and polymerized to form thearubigins and theabrownins, thereby reducing the TF content after SD. However, the TF content after SD was higher in IGT and IJT than that after FD. Therefore, drying temperature is an important factor affecting the quality of IGT. As temperatures exceed 50°C, the concentration of catechins decreases and the loss of catechins is about 4% heating to 90°C (23). It indicates that the oxidation of catechins is accelerated by the thermal effect of SD compared with the lower treatment temperature of FD, which result in increased content of TFs. Under thermal processing like SD, catechins are easy to be oxidized and polymerized into TFs (23). Although they are still flavonoids, the bioactivity and the sensory quality have changed. To better maintain the quality attributes of three raw tea, FD is more preferable for instant tea production to preserve the flavonoids compositions.

### 3.3. Characterization of the chemical composition of instant teas made from different raw tea materials

Selection of suitable raw tea materials is essential to produce high quality instant tea, because of the differences in biochemical compositions in different tea plant varieties, growing regions, and harvest seasons (8, 24), as well as the different leaching rates of the various biochemical components from the leaves during infusion (25). The biochemical composition of the green, black and jasmine tea used here are shown in Supplementary Table S1. GT had the highest content of total polyphenols, amino acids, and caffeine, followed by JT and BT. The GC concentration was highest in BT, followed by JT and GT. The most abundant catechin in BT was EC (2.34 mg/g) and that in GT and JT were EGCG (82.32 and 59.70 mg/g, respectively). The content of ester catechins and non-ester catechins in BT was 2.38 and 5.59 mg/g respectively, a ratio of 1:2.35. The ester/non-ester ratio in GT was 1.9:1 and that of JT was 2.1:1. The lower catechin content of BT than those of GT and JT indicates that the catechins in (fermented) BT are more extensively oxidized into high molecular weight tea pigments. BT contained the highest content of the four TFs (2.69 mg/g, or 0.27%), an indicator of superior quality to bulk black tea (~0.2% TFs). JT was the second highest, at 0.33 mg/g of TFs and GT was the lowest at 0.163 mg/g. The low TF content in jasmine and green tea could be related to slow catechin oxidation under hot working conditions during processing.

In total, 878 primary metabolites (Supplementary Figure S2A) and 565 flavonoids (secondary metabolites; Supplementary Figure S2B) were detected in the tea samples and displayed in heatmaps. Amino acids and derivatives accounted for 43.05% of the metabolites, followed by lipids (20.27%), organic acids (15.26%), and phenolic acids and tannins (0.69%) (Supplementary Figure S2A1). Flavonoids accounted for 93.32% of the polyphenols, followed by tannins (7.16%) and phenolic acids (0.35%) (Supplementary Figure S2A2). Lipids were abundant in raw tea, but decreased in both FD and SD instant tea, which can be attributed to their low water solubility (Supplementary Figure S2A1). Other primary metabolites were essentially retained in instant tea (Supplementary Figures S2A,B). Raw GT contained more flavonoids, and more was retained in IGT than in IJT and IBT (Supplementary Figure S2B).

**Figure 2:**
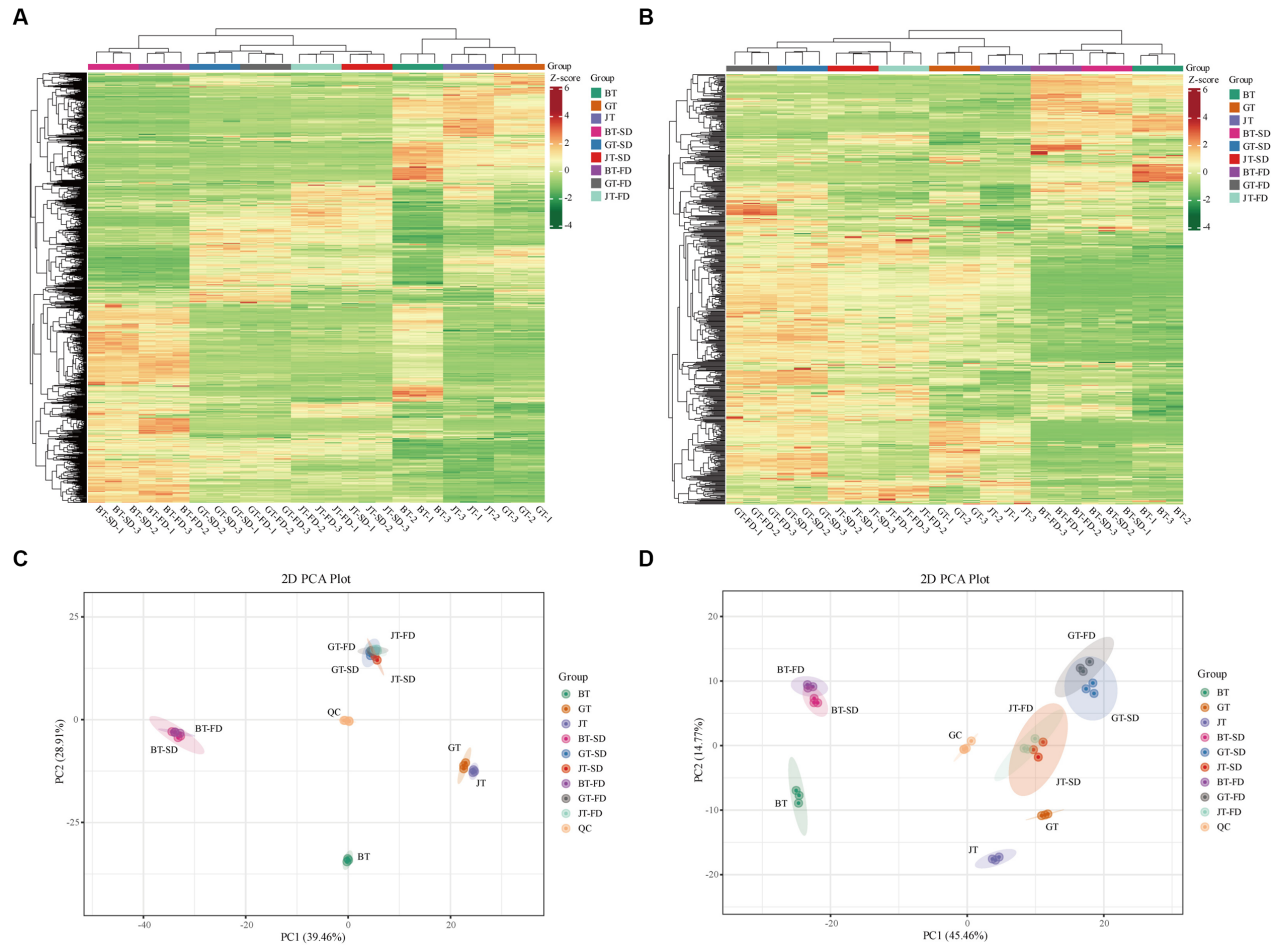
The cluster analysis and principle compound analysis of the metabolites in raw tea materials and instant tea. **(A,C)** Primary metabolites. **(B,D)** Flavonoids.

The cluster analysis of primary metabolites (Figure 2A) showed that the raw teas had marked compositional differences. Their clusters were well separated. There was greater similarity between SD and FD instant teas of each tea variety than among the three varieties of raw tea (Figure 2A). The mixed quality control samples were clustered in the center of the principal component analysis (PCA) score plot, which indicated that the tea extraction process and LC-MS analysis were consistent and reliable. In addition, in the PCA score plot, GT and JTwere closely clustered, and GT-FD, GT-SD, JT-FD and JT-SD were closely clustered (Figure 2C). This is to be expected, as jasmine tea is made from green tea leaves with added jasmine flowers to impart a flower-like aroma. Essentially, SD and FD had little effect on the primary metabolite composition of IGT and IJT. However, BT was not only well separated from GT, JT, GT-FD, GT-SD, JT-FD and JT-SD, but also from BT-SD and BT-FD (Figure 2C). SD and FD significantly changed the primary metabolite composition of IBT from that of raw BT.

In contrast to the primary metabolites, the differences in flavonoids content (secondary metabolites) among the three tea varieties was relatively large (Figure 2B) compared with the differences in primary metabolites. Raw black, green and jasmine tea were similar to their corresponding instant teas (Figure 2B). The close clustering of SD and FD instant tea flavonoids compositions of the three varieties of tea was similar to that of primary metabolites. In the PCA score plot, GT and JT were separated by 45.46% on PC1and 14.77% on PC2 (Figure 2D), but the separation was smaller than that from BT. The PC1 separation (45.46%) clearly separated BT and IBT from both green and jasmine tea and their corresponding instant teas (Figure 2D), indicating that the flavonoids composition can be used to differentiate black tea from green tea and jasmine tea, whereas the primary metabolite composition cannot.

During its original processing, raw green tea is steamed to inactivate endogenous enzymes, such as polyphenol oxidase (PPO) and peroxidase (POD). In contrast, black tea is not steamed, so PPO and POD can catalyze marked changes in flavonoids composition, transforming catechins (flavan-3-nols) into tea pigments (TFs, thearubigins, and theabrownins) (26, 27). To improve understanding of the mechanism of quality changes to instant tea resulting from SD and FD, subsequent work should focus on changes in flavonoids composition.

### 3.4. Comparison of the flavonoid composition of the different instant teas

OPLS-DA modeling was applied to the LC-MS datasets to determine which metabolites were significantly changed during the production of instant tea from different raw tea materials. After selecting the differential variables by removing the irrelevant differences, a score map of the three different raw teas was constructed (Supplementary Figure S3A). Each tea variety was clearly separated from the others, with the BT triplicate samples all grouped toward the upper right, the GT triplicate samples all grouped toward the upper left and the JT triplicate samples grouped toward the lower region. The groupings and separations were similar in the maps of the BT-SD, GT-SD and JT-SD instant teas (Supplementary Figure S3B), as well as the BT-FD, GT-FD and JT-FD instant teas (Supplementary Figure S3C). The OPLS-DA score maps showed wider separations among BT, GT and JT than was obtained from the PCA score plot.

The above OPLS-DA models were then used to construct an S-plot (Supplementary Figures S3D–F), to provide a graphical projection of specific compounds. Generally, metabolites that are far from the plot origin contribute more to the separation among different samples, and the metabolites marked as red dots at the lower left and upper right corners are significant, with variable importance in projection (VIP) values exceeding 1. In total, there were 309, 322, and 326 significantly different metabolites contributing to the separations among raw tea, SD and FD samples, respectively. Compared with PCA, OPLS-DA magnified the separation between groups, which facilitates the discovery of differential metabolites (28). These results indicate that OPLS-DA of flavonoids can be used to differentiate the three different tea varieties, either as raw tea materials or as instant teas.

**Figure 3:**
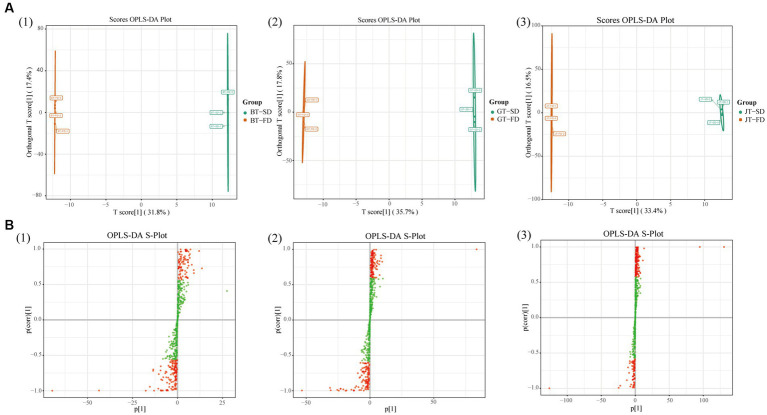
Multivariate statistical analysis of non-volatile compounds under different drying methods. Score scatter plots for the OPLS-DA models **(A)**: BT-SD vs. BT-FD; GT-SD vs. GT-FD; JT-SD vs. JT-FD; S-plots of the OPLS-DA models **(B)**: BT-SD vs. BT-FD; GT-SD vs. GT-FD; JT-SD vs. JT-FD.

**Figure 4:**
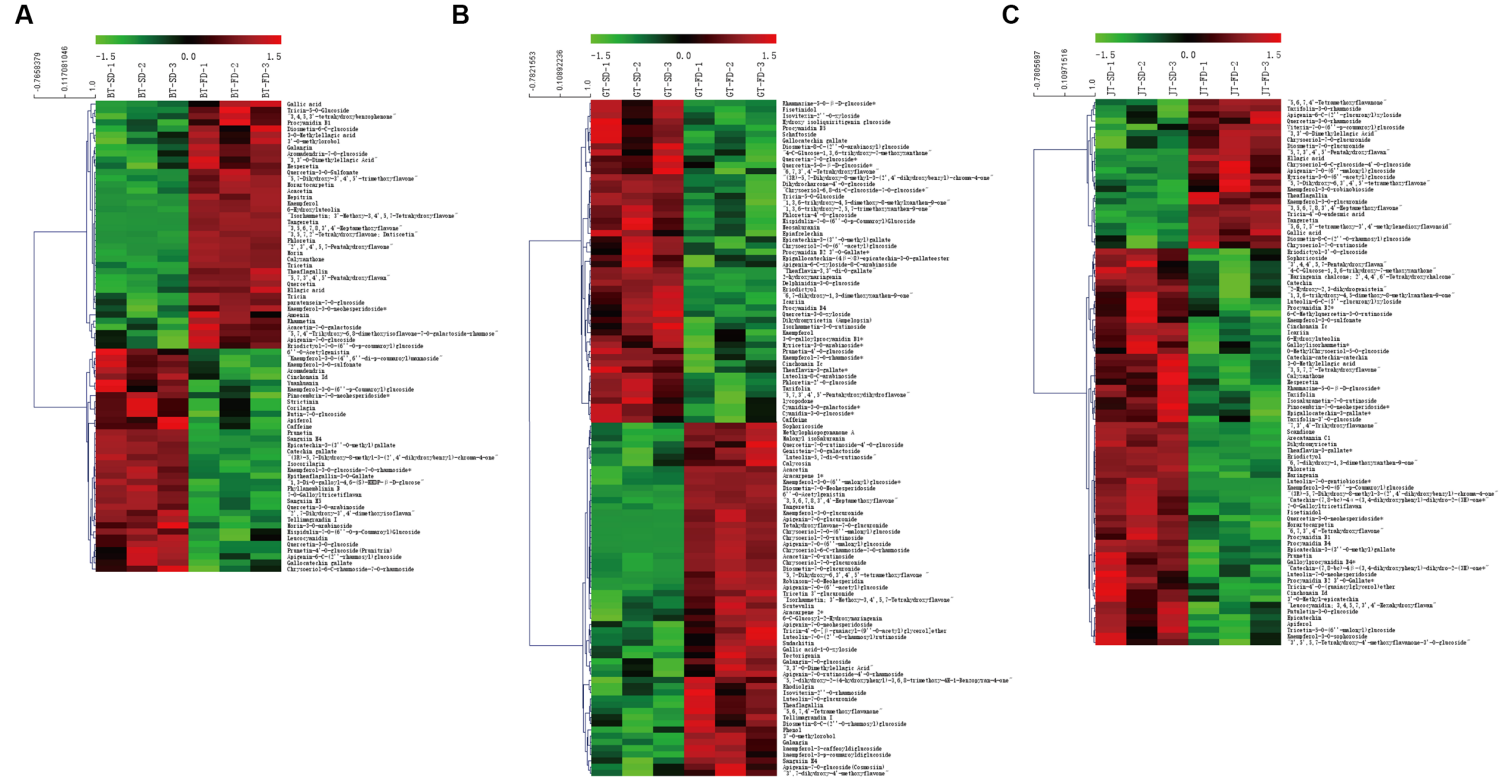
Heatmaps of key aroma compounds with VIP > 1 and *P* < 0.05 for the OPLS-DA models. Heatmaps of key aroma compounds with VIP > 1 and P < 0.05 for the OPLS-DA models. **(A)**: BT-SD vs. BT-FD; **(B)**: GT-SD vs. GT-FD; **(C)**: JT-SD vs. JT-FD.

SD and FD had significantly different effects on the flavonoid composition of black tea (Figure 3A1). BT-SD was separated from BT-FD. In addition, an S-plot was used to screen the differential metabolites between BT-SD and BT-FD, using VIP value analysis (Figure 3B1), and 224 metabolites with VIP > 1 were found. Of these, 76 differential metabolites with VIP > 1 and *p* < 0.05 were found between BT-SD and BT-FD (Figure 4A). Heatmap analysis characterized the relative content of the differential metabolites, of which 36 were more abundant in SD black tea and 40 were more abundant in FD black tea. Compared with FD, SD effectively protected the GCG (decrease), CG (decrease) and isoquercitrin (decrease) in black tea from being hydrolyzed to gallic acid (increase) and quercetin (increase).

Similar results were obtained by comparing GT-SD with GT-FD and JT-SD with JT-FD. Comparing the flavonoids in green tea, 244 metabolites with VIP > 1 were found by OPLS-DA and 109 metabolites with VIP > 1 and *p* < 0.05 were recognized as differential metabolites between GT-SD and GT-FD (Figures 3A2,B2), of which 52 were more abundant in SD green tea and 57 were more abundant in FD green tea (Figure 4B). Compared with FD, SD effectively promoted TFDG (decrease), GCG (decrease), and TF-3-G (decrease) in IGT. Comparing the flavonoids in jasmine tea, 239 metabolites with VIP > 1 were recognized by OPLS-DA and 88 metabolites with VIP > 1 and *p* < 0.05 were recognized as differential metabolites between JT-SD and JT-FD (Figures 3A3,B3), of which 64were more abundant in SD green tea and 24 were more abundant in FD green tea (Figure 4C). Compared with FD, SD effectively protected TF-3-G (decrease), EC (decrease), C (decrease), and EGCG (decrease) in raw JT from being hydrolyzed to GC (increase).

The changes in TFDG, and TF-3-G in GT and JT confirmed that the oxidation of catechins was accelerated by the high temperature of SD compared with the lower treatment temperature of FD, resulting the increase of TFs. FD is more suitable for instant tea production from green and jasmine tea. Important quality attributes for BT are the content of tea pigments and aroma (29). The hydrolysis of some flavone glycosides influences the appearance, color and aroma of black tea (6). Therefore, unlike GT and JT, changes in the chemical composition of BT after SD treatment appear to improve its sensory quality, compared with FD treatment.

### 3.5. Effect of SD and FD treatments on volatile profiles of instant teas

A total of 177 volatile compounds were detected in the three raw tea materials and their corresponding instant teas, permitting analysis of the influence of chemical compositional changes on the overall flavor. These compounds included 35 alcohols, 28 aldehydes, 39 ketones, 39 esters, 28 alkenes, six heterocyclic compounds, and two acids (Supplementary Figure S4). Comparing the three raw tea varieties, JT was clearly the most abundant in volatile compounds, followed by BT, with GT the least abundant (Supplementary Figure S4A). The same order of volatile compound abundance applied to their SD and FD instant teas. The main components in the three tea varieties were different, with aldehydes (36.06%) the major compound class in BT, ketones (37.09%) in GT, and esters (51.53%) in JT (Supplementary Figure S4B). This indicates that during the scenting process of raw GT with jasmine flowers, raw GT absorbs the floral aroma, thus the content of aroma compounds is much more abundant than BT and GT. For instant tea, using scented teas like jasmine tea can facilitate the production of fragrant tea.

After drying, the main volatile components in the JT-SD and JT-FD were still esters (48.31 and 50.65%, respectively), those in BT-SD were still aldehydes (36.42%), and those in GT-FD were still ketones (37.30%) (Supplementary Figure S4B). However, the main volatile components in BT-FD were alcohols (34.93%), and those in GT-SD were esters (34.21%). Although both SD and FD teas contained less esters and alcohols than the corresponding raw teas, FD retained more aroma compounds than SD for all three tea varieties. The low temperature of FD reduces evaporation and degradation of aroma compounds (21), compared with the high temperature of the SD tower, i.e., FD is more suitable for producing scented instant teas.

After literature and database matching, 49 aroma compounds were identified (Supplementary Table S2) and OAV analysis was used to compare the contribution of each compound to the overall tea aroma. The 15 most abundant volatiles were benzyl acetate (0.03–35.55 μg/g), methyl benzoate (0.00–8.94 μg/g), linalool (0.01–8.45 μg/g), benzaldehyde (0.25–7.88 μg/g), (*E*,*E*)-2,4-heptadienal (0.22–6.69 μg/g), methyl salicylate (0.05–5.56 μg/g), phenylethyl alcohol (0.14–4.18 μg/g), geraniol (0.04–3.90 μg/g), neral (0.01–3.90 μg/g), 3,5-octadien-2-one (0.06–3.73 μg/g), benzeneacetaldehyde (0.04–3.27 μg/g), benzyl alcohol (0.17–2.83 μg/g), (*Z*)-3-hexenyl acetate (0.01–2.54 μg/g), indole (0.01–1.56 μg/g) and (*E*)-2-octenal (0.02–1.03 μg/g) (Supplementary Table S2). Apart from benzyl alcohol, these major volatiles were all more abundant in the raw tea materials than in the corresponding instant teas and were more abundant in FD teas than SD teas, further elucidated that FD reduced evaporation and degradation of aroma compounds compared with SD.

The main volatiles have different odor thresholds, so their concentrations in tea infusions do not necessarily match their relative abundances. To compare the contribution of each compound to the overall tea aroma, GC–MS combined with OAV analysis identified nine compounds with rOAVs >1 (Table 3), namely *trans*-*β*-damascenone, linalool, benzeneacetaldehyde, geraniol, nonanal, methyl salicylate, safranal, neral, and (*Z*)-3-hexenyl acetate. The rOAVs of *trans*-*β*-damascenone (40.00–1200.00), linalool (0.36–768.82), benzeneacetaldehyde (0.06–16.39), geraniol (0.03–10.42), nonanal (0.55–4.89) and methyl salicylate (0.00–2.78) were the highest of the nine compounds, indicating that they are major contributors to the aroma of the three tea varieties and their corresponding instant teas.

**Table 3:** OAV analysis of volatiles in the instant teas with different tea varieties and drying methods.

No	Compound	Odor description^a^	Odor type^b^	Odor threshold in water (μg/L)^c^	RI	rOAV								
						BT^¶^	GT^¶^	JT^¶^	BT-SD^§^	GT-SD^§^	JT-SD^§^	BT-FD^§^	GT-FD^§^	JT-FD^§^
Alcohols														
1	Linalool	Floral, sweet	Floral	0.22	1087.902	237.27	45.00	768.82	1.84	0.36	24.41	90.75	22.70	161.84
2	Geraniol	Rose-like, sweet, honey-like	Floral	7.5	1274.734	10.42	0.52	0.87	0.09	0.03	0.04	1.20	0.19	0.21
Aldehydes														
3	Benzeneacetaldehyde	Floral, rose, cherry-like	Floral	4	1033.525	16.39	1.28	0.23	5.32	0.50	0.41	1.85	0.27	0.06
4	Nonanal	Fatty and herbal smell	Floral	1.1	1115.703	4.89	1.95	1.95	0.82	1.02	0.76	0.95	0.55	1.62
5	Safranal	Woody, spicy, phenolic	Woody	3	1237.296	1.66	1.07	1.14	0.56	0.23	0.24	0.23	0.21	0.21
6	Neral	Lemon	Fruity	53	1264.188	1.47	0.02	0.01	0.00	0.00	0.00	0.02	0.00	0.00
Ketones														
7	*trans*-*β*-Damascenone	Rose, honey	Floral	0.002	1392.773	1200.00	290.00	300.00	137.50	47.50	40.00	325.00	87.50	82.50
Esters														
8	(*Z*)-3-Hexenyl acetate	Green, banana	Green	31	1004.607	0.05	0.29	1.64	0.00	0.01	0.01	0.02	0.02	0.13
9	Methyl salicylate	Fresh, faint gingery, grass and milky	Green	40	1162.976	1.45	0.17	2.78	0.01	0.00	0.09	0.44	0.08	0.65

^a, b, c^ Odor descriptions, types and thresholds were found in the database (http://www.flavornet.org/) and literatures (18, 30). ^¶^rOAVs of raw tea materials were converted based on a tea to water ratio of 1: 50 for brewing. ^§^rOAVs of the instant teas were converted based on a tea to water ratio of 1:200 for brewing.

Of these aroma compounds, *trans*-*β*-damascenone has the lowest odor threshold (0.002 μg/L) and its rOAV in BT is higher than those in GT and JT, contributing a strong “rose” fragrance and the main contributor to the aroma of black tea (31). Geraniol, nonanal, and neral are major floral and fruity volatiles in black tea (31, 32), and in this study their rOAVs in black teas were higher than those in jasmine and green teas. Safranal contributes the woody aroma characteristic of souchong and congou black tea (33), and it had the highest rOAV in BT, followed by JT, GT and their instant teas. This indicated that these four floral and fruity compounds were well-retained in BT, JT and GT, but efficiently released from the instant tea. Again, FD teas retained much more of these volatiles than SD teas, further evidence that FD reduces evaporation and degradation of aroma compounds, compared with SD.

Linalool has a low odor threshold (0.22 μg/L) and a sweet floral note and its rOAVs in JT and IJT were higher than those in black and green teas (32). Linalool has the highest relative abundance in jasmine tea, making it the main flavoring component (34), indicating that scenting with jasmine flowers increases the content of linalool well above that in black tea. Methyl salicylate, another jasmine flower component is well-absorbed by the tea leaves (34), has the aroma of holly leaves and mint and its content was higher in JT, followed by BT and their instant teas. (*Z*)-3-Hexenyl acetate is also present in jasmine tea and its content was higher in JT and its instant teas (Table 3). Therefore green tea absorbs linalool, methyl salicylate and (*Z*)-3-hexenyl acetate released from jasmine flowers during scenting, and this greatly strengthened the floral aroma of jasmine tea, however, their content decreased during instant tea preparation, weakening its floral aroma.

In general, BT and its instant teas contained the most floral and fruity aroma compounds, especially *trans*-*β*-damascenone, geraniol, nonanal, and neral. JT and its instant teas contained the much more floral linalool and fresh methyl salicylate and (*Z*)-3-hexenyl acetate. GT and its instant teas contained far less aroma compounds than BT and JT. Instant tea preparations contained less of these aroma compounds than the raw teas, but FD reduced evaporation and degradation of aroma compounds compared with SD.

### 3.6. Correlation analysis between flavonoid content and volatile profile of different raw tea materials

Since flavonoids have been confirmed to contribute to tea aroma (6, 35) and OPLS-DA can differentiate between the aroma profiles of IBT, IGT and IJT, changes in flavonoid content between SD and FD samples may contribute to the aroma of the instant teas. Differential metabolite analysis with VIP values and fold-change was used to generate volcano plots of the increased and decreased differential metabolites (Supplementary Figure S5).

**Figure 5:**
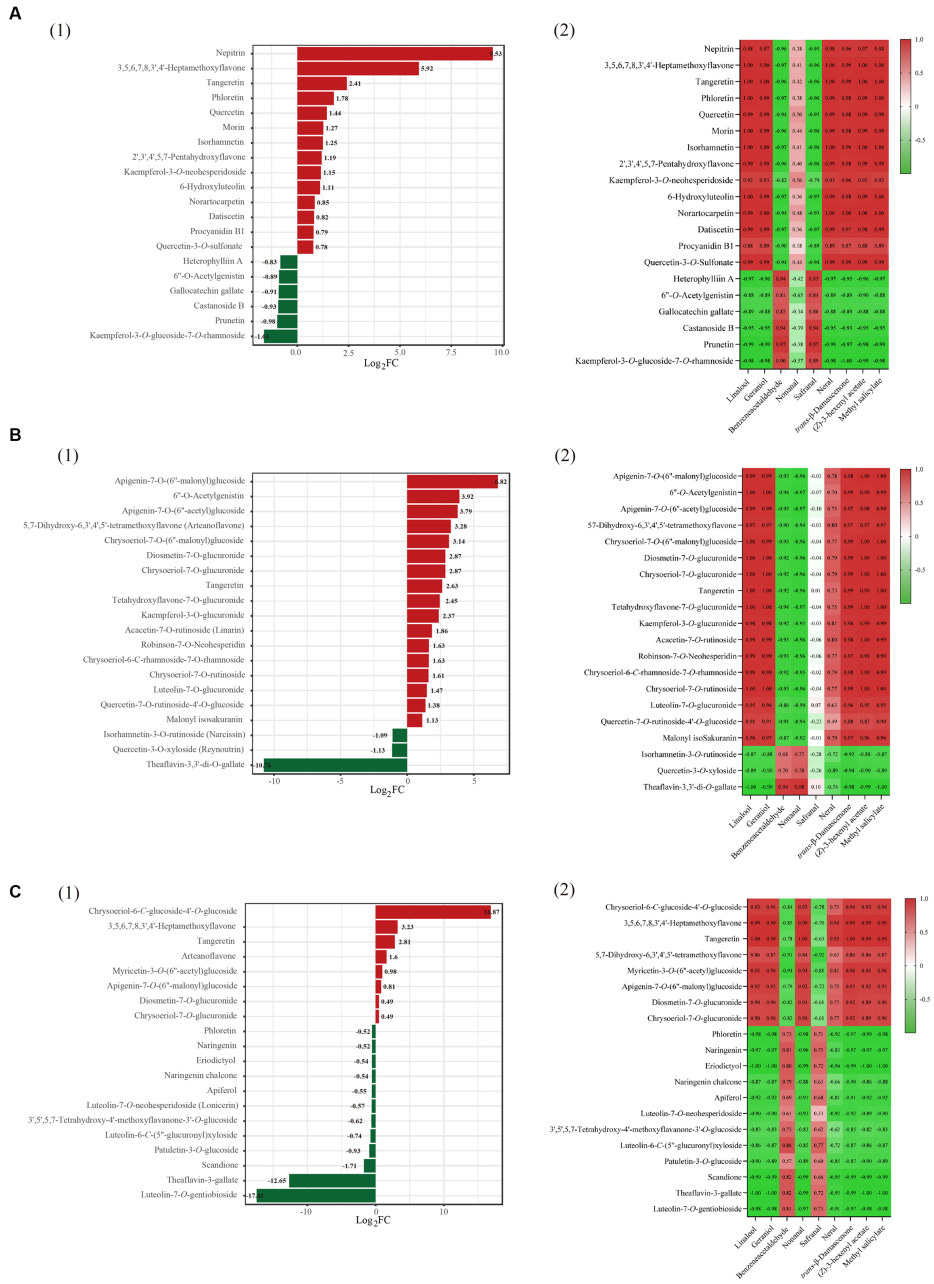
Multivariate statistical analysis of flavonoids and volatile compounds in three tea varities. Group **(A)** refers to instant black tea, group **(B)** refers to instant green tea, and group **(C)** refers to instant jasmine tea. Dynamic distribution map of differences in the content of the top 10 substances, up and down, in three instant teas (SD vs. FD) (group 1); heatmap of the pearson correlation coefficient of the volatiles (rOAV > 1) and the content of the top 10 substances (group 2).

Comparing BT-SD and BT-FD, found 76 significant differential flavonoids, of which 40 increased and 36 decreased, respectively (Supplementary Figure S5A). The above limits of fold-change found 20 significant differential metabolites between FD and SD instant black teas (Figure 5A1), namely, 6 glycosylated flavonoids, 12 flavones and 2 flavon-3-ols (procyanidin B1 and GCG). Of these, 14 were more abundant, including 10 non-glycosylated flavonoids (3,5,6,7,8,3,4-heptamethoxyflavonenepitrin, tangeretin, phloretin, quercetin, morin and isorhamnetin, 2′,3′,4′,5,7-pentahydroxyflavone, 6-hydroxyluteolin, norartocarpetin, datiscetin) and six were less abundant, including three glycosylated flavonoids (heterophylliin A, Castanoside B and kaempferol-3-*O*-glucoside-7-*O*-rhamnoside).

To statistically analyze the relationship between the volatiles with rOAV>1 and the content of the top ten differential flavonoids, Pearson’s correlation coefficient was calculated and used to generate a correlation heatmap (Figure 5A2). Top ten abundant non-glycosylated flavonoids positively were correlated with the contents of six aroma compounds, linalool, geraniol, nerol, *trans*-*β*-damascenone, (*Z*)-hexenyl acetate, and methyl salicylate. Three glycosylated flavonoids, less abundant ones, were negatively correlated with the contents of the above six aroma compounds. This suggests that the less-abundant glycosylated flavonoids are hydrolyzed or degraded during drying stage of instant tea, resulting in the loss of glycosylated aroma compounds and gains of the corresponding non-glycosylated flavonoids. During the withering stage of congou black tea processing, the content of flavones, flavonols and flavonoid polymers (mainly consisting of TFs) increased, whereas hydrolysis of flavonoid glycosides mainly occurred during the rolling stage (35). The thermal stability of flavonoids influenced their final content, and glycosylated flavonols are less sensitive to heat treatment than aglycon ones (35). Drying stage therefore is crucial for the final aroma of instant tea.

Comparing GT-SD and GT-FD found 109 significant differential flavonoids, of which 57 increased and 52 decreased (Supplementary Figure S5B). Of the 20 significantly different flavonoids, there were 17 glycosylated flavonoids, 2 flavones and 1 flavon-3-ol (TFDG). Eighteen flavonoids were more abundant, including three non-glycosylated flavonoids (6”-*O*-acetylgenistin, arteanoflavone, tangeretin), and two flavonoids were less abundant including two glycosylated flavonoids (narcissin and reynoutrin) (Figure 5B1). From correlation analysis (Figure 5B2), three more abundant non-glycosylated flavonoids were positively correlated with the contents of linalool, geraniol, nerol, *trans*-*β*-damascenone, (*Z*)-hexenyl acetate, methyl salicylate and neral. Two glycosylated flavonoids, less abundant ones, were negatively correlated with the contents of the above seven aroma compounds.

Comparing JT-SD and JT-FD found 88 significant differential flavonoids, of which 24 increased and 64 decreased (Supplementary Figure S5C). Of the 20 significantly different flavonoids, there were 10 glycosylated flavonoids, nine flavones and one flavon-3-ol (TFDG). Eight flavonoids were more abundant including three non-glycosylated flavonoids (3,5,6,7,8,3″,4- heptamethoxyflavone, tangeretin and arteanoflavone), and 12 flavonoids were less abundant, including five glycosylated flavonoids (lonicerin, 3′,5′,5,7-tetrahydroxy-4′-methoxyflavanone-3’-*O*-glucoside, luteolin-6-*C*-(5″-glucuronyl) xyloside, patuletin-3-*O*-glucoside and luteolin-7-*O*-gentiobioside) (Figure 5C1). From correlation analysis, three increased non-glycosylated flavonoids were positively correlated with the contents of linalool, geraniol, nerol, *trans*-*β*-damascenone, (*Z*)-hexenyl acetate, methyl salicylate, nonanal and neral (Figure 5C2). Five decreased glycosylated flavonoids were negatively correlated with the contents of the above eight aroma compounds.

Many aroma components exist in tea in the form of glycoside precursors, including monoterpene alcohols (linalool, linalool oxides, and geraniol), aromatic alcohols (benzyl alcohol and phenylethanol), and some non-alcoholic volatile aromas such as benzaldehyde and *β*-damascenone (36, 37). These glycosidically bound volatile aromas take more steps than glycosidically bound alcoholic volatiles to release free volatile aromas (38). Besides, a strong acidic condition (pH 2.0) with a high temperature (90°C) favors the hydrolysis of glycosidic bonds (38). From Table 3 we can see that FD instant teas contains more alcohol aromas including linalool and geraniol. From the above results and Supplementary Table S1 we can see that gallic acid content increase from raw tea to drying stage when flavonoids like EGCG and ECG decrease. Flavonoids degrade at different rates during the drying of black tea, because of their thermal instability, for example, luteolin-7-*O*-glucoside is more sensitive to heat treatment than its aglycone form luteolin (39). Anthocyanins and procyanidin A3 are especially unstable and may also be thermally degraded by oxidation and cleavage of covalent bonds (39, 40). This may indicate that the high temperature (over 90°C) in SD accelerates the hydrolysis of flavonoids and glycoside precursors of monoterpene alcohols, both of which form the final aroma of instant tea. Further analysis on the contributions of these changed glycosylated flavonoids to aroma is in need to facilitate instant tea drying. Besides, the correlations between glycosylated flavonoids and aroma compounds during drying of instant tea need further research. In summary, tea flavonoids change markedly during instant tea manufacturing, especially during drying, which contributes directly to the formation of flavor.

## 4. Conclusion

With an aim to elucidate the impact of drying methods and raw materials on the organoleptic and physicochemical properties of instant tea, we prepared instant teas from three tea varieties using two drying methods, with a focus on the inherent primary and secondary plant metabolites through widely targeted metabolomics. The raw tea materials and the drying methods strongly influenced the quality of instant tea. Sensory evaluation and physicochemical characterization revealed that freeze-drying (FD) can better maintain the sensory quality of instant teas made from black, green and jasmine raw tea materials. All sensory evaluation scores of FD samples were better than spray-dried (SD) instant tea. Black tea was more stable in quality changes between two drying methods. Green tea liquor color can be well maintained while its taste deteriorates much after SD. Jasmine tea liquor color can also be well maintained, while its aroma deteriorates much more after SD. Therefore, jasmine tea must be produced from FD to maintain its characteristic aroma. Chemical compositional analysis found that SD significantly decreased the abundance of the main flavor and quality components of black tea. SD promoted the formation of theaflavins (TFs) at high temperature. Widely targeted metabolomics revealed that the flavonoids composition changed markedly after drying and flavonoids analysis by OPLS-DA helped differentiate black tea from green and jasmine tea. Volatile compositional analysis found that drying to produce instant tea results in loss of tea volatile compounds, but FD retained more of the main floral and fruity compounds in the instant tea than SD. There were major compositional changes in the tea flavonoids during drying procedure of instant tea production, which contributed directly to the formation of the flavor of instant tea. These results provide an practicle method for high-quality instant tea production through choosing proper raw tea material like jasmine tea and lowering down drying temperature with non-thermal technologies like FD.

## Data availability statement

The original contributions presented in the study are included in the article/Supplementary material, further inquiries can be directed to the corresponding author.

## Author contributions

J-CJ: funding acquisition, investigation, data curation, and writing – original draft. SL: methodology, investigation, data curation, writing – original draft, and writing – review and editing. S-XQ, PT, J-XC, and Y-FC: investigation. Q-SC: writing – review and editing. J-FY: investigation and writing – review and editing. Y-QX: funding acquisition, investigation, and writing – review and editing. All authors contributed to the article and approved the submitted version.

## Footnotes

^1^ http://www.hmdb.ca
