# Supplementary material for: Widely targeted metabolomics reveals the effect of different raw materials and drying methods on the quality of instant tea
Source: Front Nutr. 2023 Oct 11;10:1236216. doi: 10.3389/fnut.2023.1236216 (PMC10600452; doi:10.3389/fnut.2023.1236216)
Supplement: Supplementary file 1 [file Data_Sheet_1.pdf]

Supplementary Material

Widely targeted metabolomics reveals the effect of different raw materials and drying methods on the quality of instant tea

Jian-Chang Jin[1, a], Shuang Liang[2, a], Shang-Xiong Qi[3], Ping Tang[4], Jian-Xin Chen[2], Quan-Sheng Chen[5], Yan-Feng Chen[6], Jun-Feng Yin[2], Yong-Quan Xu[2*]

*** Correspondence:** Yong-Quan Xu: yqx33@126.com

1 Supplementary Figures and Tables

1.1 Supplementary Figures

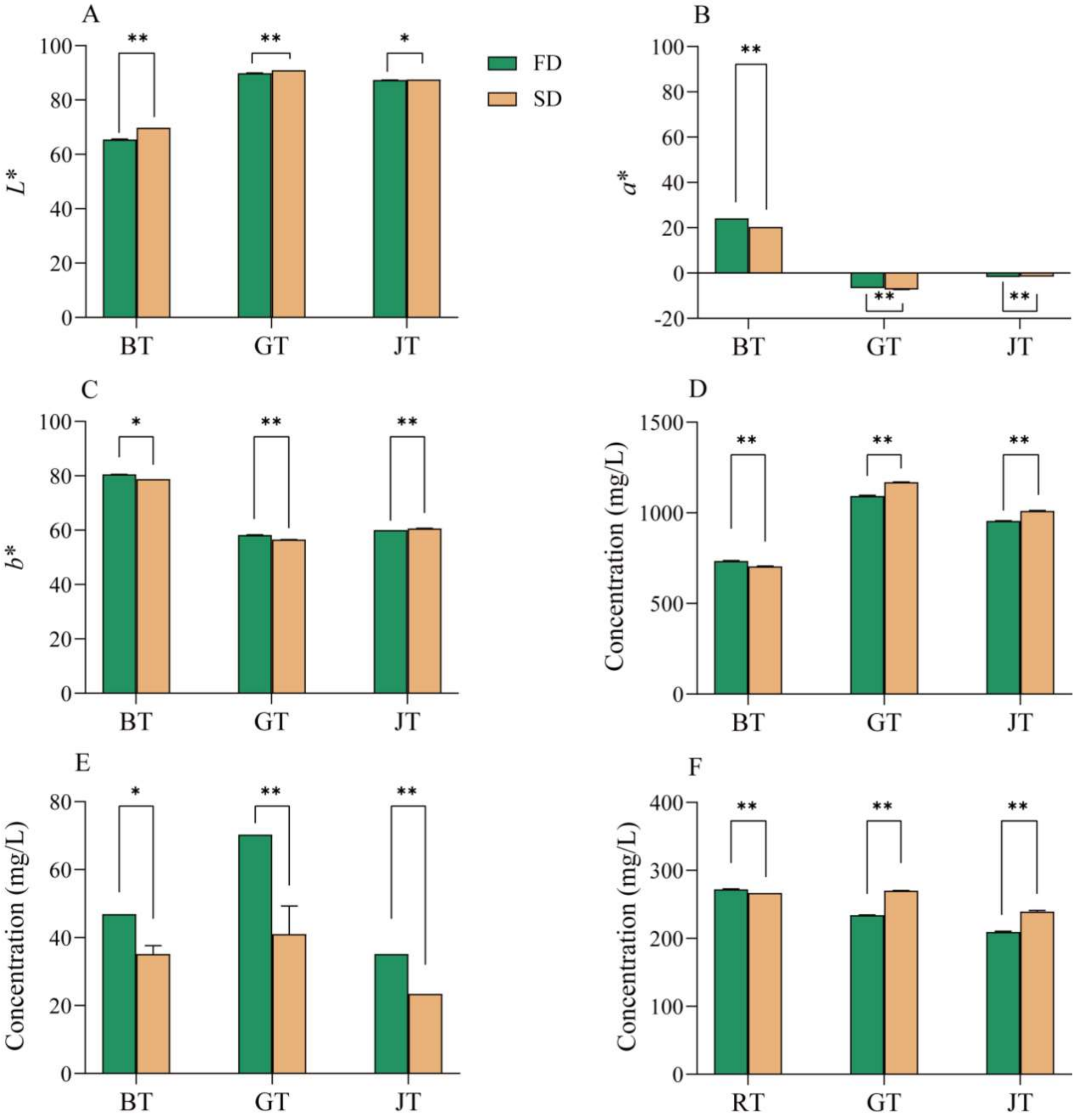

Supplementary Figure 1. Effect of spray- (SD) and freeze- (FD) drying on the color and composition of instant tea solution. (A), *L**; (B), *a**; (C), *b**; (D), tea polyphenols; (E), free amino acids; (F), caffeine. FD, freeze drying; SD, spray drying. BT, black tea; GT, green tea; JT, jasmine tea. *, indicates a significant difference ($p<0.05$); **, indicates a very significant difference ($p<0.01$).

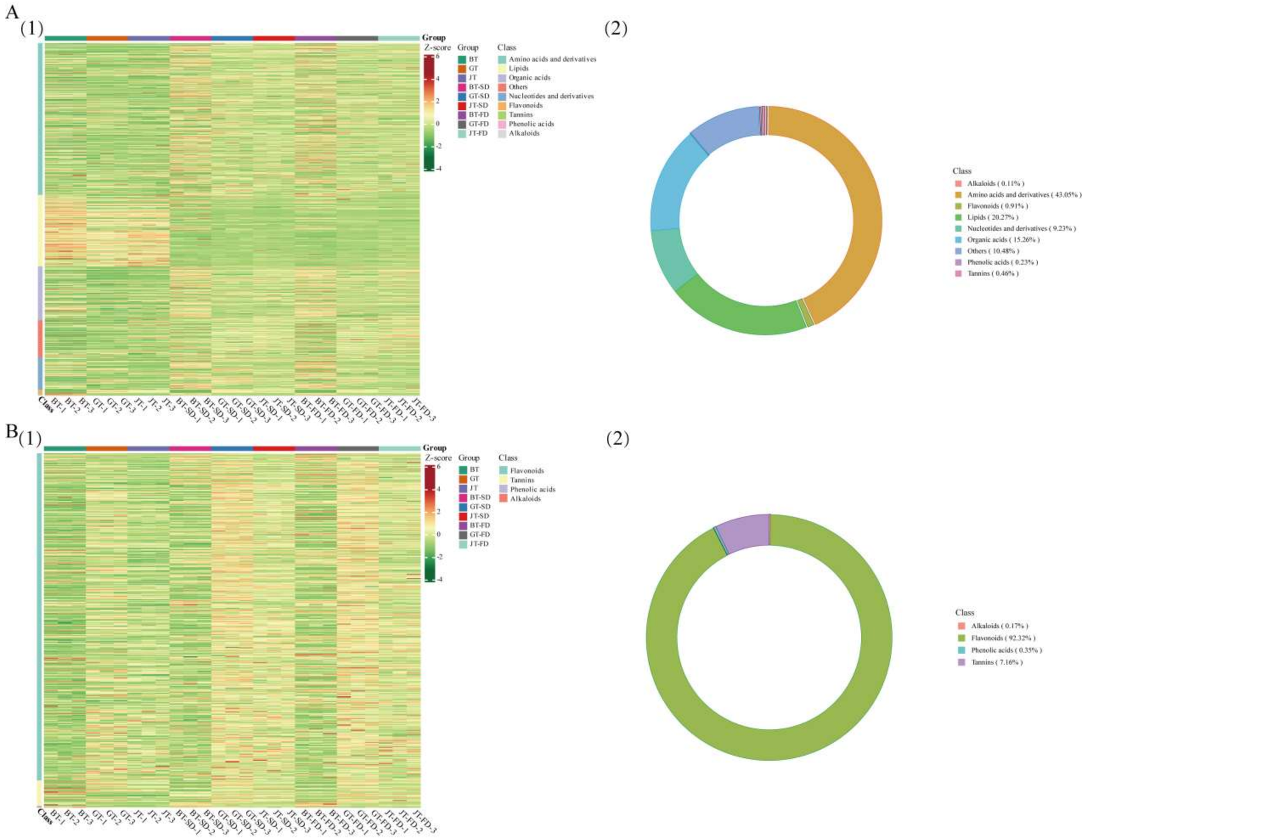

Supplementary Figure 2. Biochemical/metabolite composition of raw tea material and instant teas. (A): primary metabolites. (B): flavonoids. Nine groups of tea samples were instant teas prepared from three tea varieties, namely black (BT), green (GT) and jasmine tea (JT), using spray-drying (SD) and freeze-drying (FD).

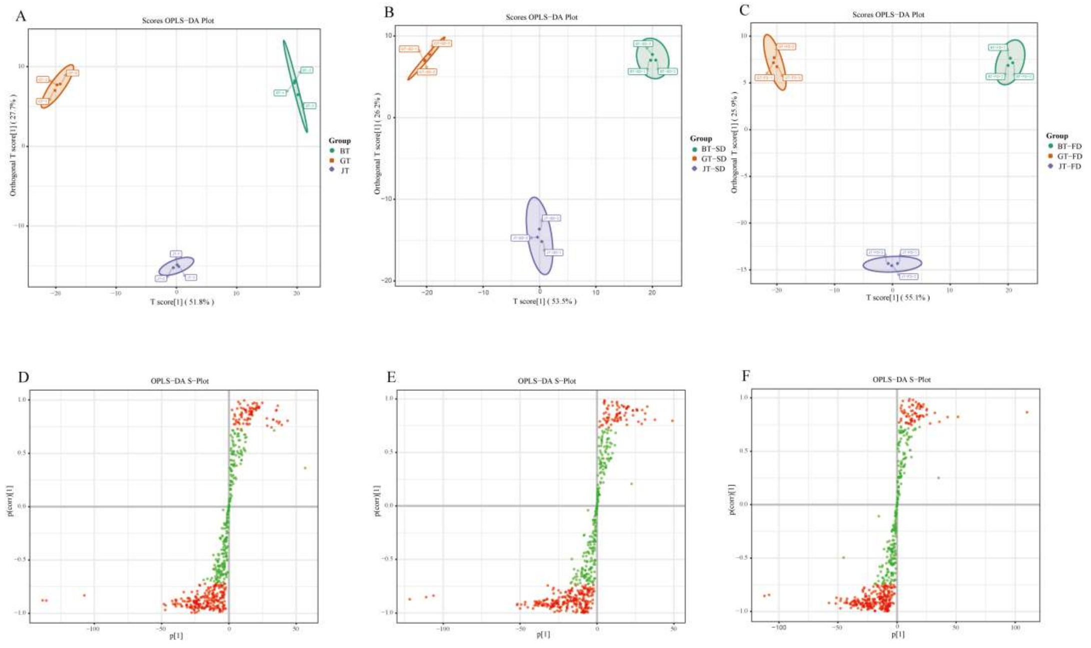

Supplementary Figure 3. Orthogonal partial least squares-discriminant analysis (OPLS-DA) of raw teas (A, D), SD teas (B, E) and FD teas (C, F). (A, B, C), score scatter plots from the OPLS-DA model; (D, E, F), S-plots of the OPLS-DA model.

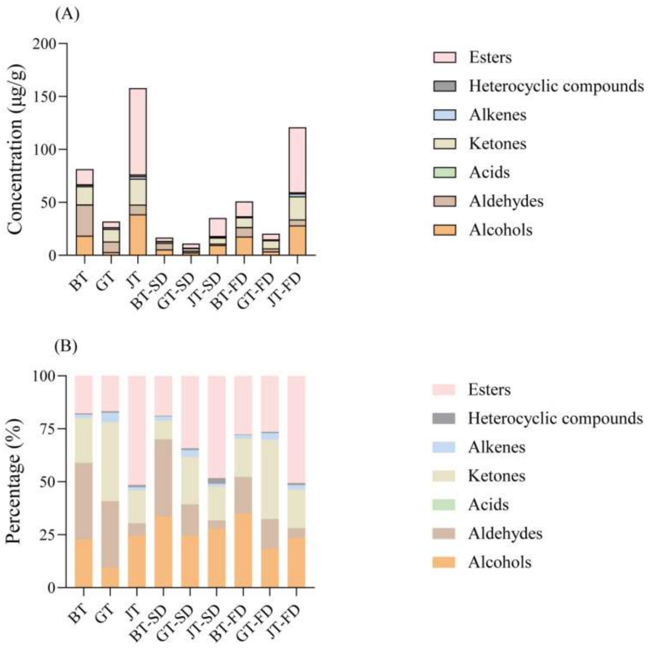

Supplementary Figure 4. Volatile compositions of three tea varieties and its instant teas. A, the concentration of different aroma compositions; B, the percentage of different aroma compositions.

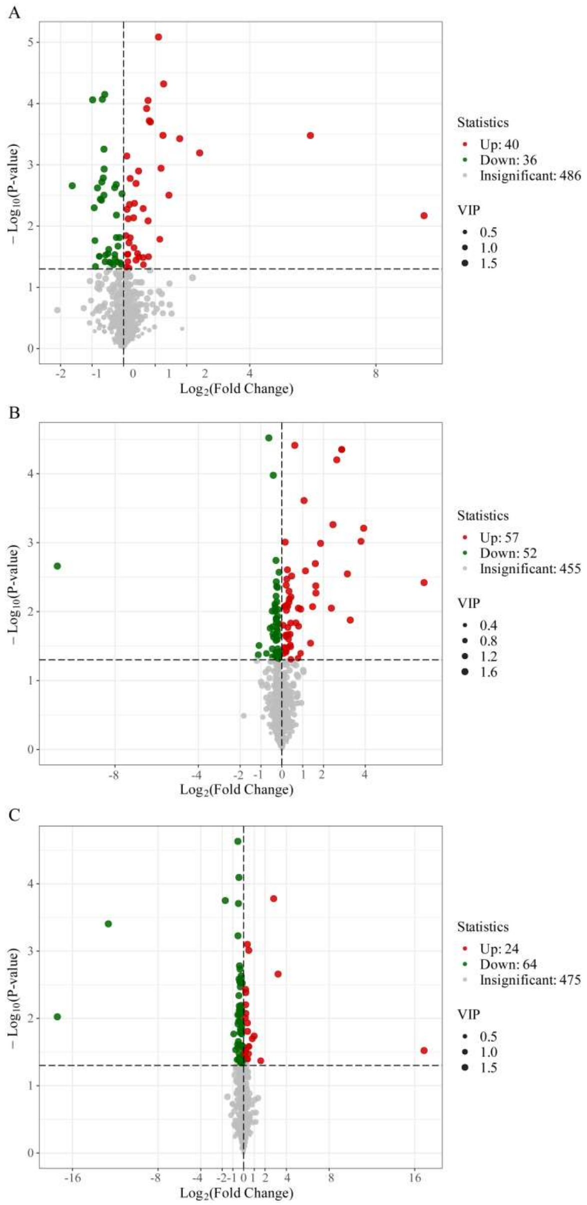

Supplementary Figure 5. Volcano plots of differential flavonoids between (A) BT-SD vs BT-FD, (B) GT-SD vs GT-FD, (C) JT-SD vs JT-FD.

1.2 Supplementary Tables

Supplementary Table 1 Chemical compositions of different raw tea materials.

Compounds	Concentration/(mg/g)		
	Black tea	Green tea	Jasmine tea
Tea polyphenols	107.978±0.353	185.277±0.294	168.960±0.177
Free amino acids	4.982±0.171	10.550±0.000	8.498±0.414
Caffeine	18.913±0.049	21.224±0.001	19.522±0.002
Gallic acid	2.142±0.001	0.599±0.007	1.055±0.006
GC	2.083±0.043	3.423±0.059	2.394±0.001
EGC	0.277±0.014	22.954±0.005	14.977±0.062
C	0.887±0.032	1.081±0.069	0.894±0.021
EGCG	0.994±0.091	82.324±0.034	59.702±0.022
EC	2.344±0.021	24.125±0.072	16.248±0.280
GCG	0.316±0.001	0.513±0.020	0.958±0.003
ECG	0.852±0.005	14.971±0.257	9.992±0.009
CG	0.219±0.004	1.290±0.064	1.289±0.012
TF	0.417±0.008	0.085±0.020	0.178±0.013
TF-3-G	0.730±0.006	0.052±0.007	0.056±0.019
TF-3'-G	0.940±0.008	0.005±0.006	0.039±0.005
TFDG	0.605±0.003	0.020±0.003	0.062±0.006

Supplementary Table 2 Odor description and concentration of volatiles in the instant teas with different tea varieties and drying methods.

No.	Compound	Odor description[a]	Odor threshold in water (µg/L)[b]	Concentration (µg/g dry weight of tea samples[*])									rOAV								
				BT	GT	JT	BT-SD	GT-SD	JT-SD	BT-FD	GT-FD	JT-FD	BT[¶]	GT[¶]	JT[¶]	BT-SD[§]	GT-SD[§]	JT-SD[§]	BT-FD[§]	GT-FD[§]	JT-FD[§]
**Alcohols**																					
1	Benzyl alcohol	Fruity, rose-like	20000	2.84	0.18	3.57	1.01	0.13	5.47	1.46	0.19	1.28	0.00	0.00	0.00	0.00	0.00	0.00	0.00	0.00	0.00
2	Linalool	Floral, sweet	0.22	2.61	0.50	8.46	0.08	0.02	1.07	3.99	1.00	7.12	237.27	45.00	768.82	1.84	0.36	24.41	90.75	22.70	161.84
3	Phenylethyl alcohol	Floral, rose-like	390	4.18	0.24	0.15	1.97	0.23	0.22	2.51	0.35	0.22	0.21	0.01	0.01	0.03	0.00	0.00	0.03	0.00	0.00
4	1-Nonanol	Fat, green	/	0.14	0.05	0.02	0.01	0.01	0.01	0.02	0.03	0.03	/	/	/	/	/	/	/	/	/
5	Terpinen-4-ol	Turpentine, nutmeg, must	0.13	0.08	0.02	0.03	0.00	0.01	0.00	0.03	0.04	0.00	0.62	0.15	0.23	0.00	0.08	0.00	0.23	0.31	0.00
6	*L*-α-Terpineol	Pleasant, floral	330	0.26	0.09	0.36	0.01	0.03	0.02	0.09	0.10	0.53	0.02	0.01	0.02	0.00	0.00	0.00	0.00	0.00	0.01
7	Nerol	Floral, fresh, citrus	49	0.19	0.02	0.04	0.01	0.01	0.01	0.10	0.03	0.04	0.08	0.01	0.02	0.00	0.00	0.00	0.01	0.00	0.00
8	Citronellol	Rose	/	0.01	0.01	0.00	0.00	0.00	0.00	0.15	0.01	0.01	/	/	/	/	/	/	/	/	/
9	Geraniol	Rose-like, sweet, honey-like	7.5	3.91	0.20	0.33	0.13	0.04	0.06	1.80	0.29	0.32	10.42	0.52	0.87	0.09	0.03	0.04	1.20	0.19	0.21
10	1-Dodecanol	Fat, wax	/	0.01	0.02	0.03	0.00	0.00	0.03	0.00	0.01	0.02	/	/	/	/	/	/	/	/	/
11	Nerolidol	Floral, green, citrus, waxy	250	0.13	0.04	0.07	0.01	0.01	0.03	0.02	0.02	0.05	0.01	0.00	0.01	0.00	0.00	0.00	0.00	0.00	0.00

12	T-Muurolol	Herb, weak spice	/	0.00	0.00	0.03	0.00	0.00	0.02	0.00	0.00	0.03	/	/	/	/	/	/	/	/	/
13	Phytol	Floral, balsam, powdery, waxy	640	0.00	0.00	0.00	0.00	0.00	0.00	0.00	0.00	0.00	0.00	0.00	0.00	0.00	0.00	0.00	0.00	0.00	0.00
**Aldehydes**																					
14	Benzaldehyde	Honey, floral	350	7.88	2.18	2.65	0.56	0.26	0.37	3.58	0.80	1.02	0.45	0.12	0.15	0.01	0.00	0.01	0.05	0.01	0.01
15	(*E*,*E*)-2,4-Heptadienal	Nut, fat	10000	6.70	5.44	1.99	0.29	0.49	0.23	0.35	0.69	0.35	0.01	0.01	0.00	0.00	0.00	0.00	0.00	0.00	0.00
16	Benzeneacetaldehyde	Floral, rose, cherry-like	4	3.28	0.26	0.05	4.26	0.40	0.33	1.48	0.22	0.05	16.39	1.28	0.23	5.32	0.50	0.41	1.85	0.27	0.06
17	(*E*)-2-Octenal	Green, nut, fat	/	1.03	0.71	0.23	0.02	0.06	0.04	0.15	0.31	0.09	/	/	/	/	/	/	/	/	/
18	Nonanal	Fatty and herbal smell	1.1	0.27	0.11	0.11	0.05	0.06	0.04	0.05	0.03	0.09	4.89	1.95	1.95	0.82	1.02	0.76	0.95	0.55	1.62
19	Safranal	Woody, spicy, phenolic	3	0.25	0.16	0.17	0.33	0.14	0.14	0.13	0.14	0.13	1.66	1.07	1.14	0.56	0.23	0.24	0.21	0.23	0.21
20	Decanal	Soap, orange peel, tallow	0.0001	0.04	0.01	0.01	0.01	0.01	0.01	0.00	0.00	0.00	400.00	100.00	100.00	100.00	100.00	100.00	0.00	0.00	0.00
21	Neral	Lemon	53	3.91	0.05	0.02	0.01	0.01	0.01	0.24	0.03	0.03	1.47	0.02	0.01	0.00	0.00	0.00	0.02	0.00	0.00
**Ketones**																					
22	3-Octen-2-one	Nut, crushed bug	/	0.37	0.09	0.02	0.01	0.02	0.02	0.07	0.05	0.00	/	/	/	/	/	/	/	/	/
23	3,5-Octadien-2-one	Fruit, fat, mushroom	/	3.74	1.74	0.53	0.03	0.09	0.06	0.89	1.27	0.44	/	/	/	/	/	/	/	/	/
24	5-Octanolide	Peach	/	0.20	0.01	0.02	0.08	0.04	0.04	0.09	0.04	0.03	/	/	/	/	/	/	/	/	/

25	γ-Nonalactone	Coconut, peach	/	0.17	0.09	0.12	0.04	0.06	0.08	0.08	0.07	0.12	/	/	/	/	/	/	/	/	/
26	*trans*-β-Damascenone	Rose, honey	0.002	0.12	0.03	0.03	0.06	0.02	0.02	0.13	0.04	0.03	1200.00	290.00	300.00	137.50	47.50	40.00	325.00	87.50	82.50
27	α-Ionone	Floral, woody, violet-like	76	0.96	0.65	0.37	0.01	0.04	0.04	0.09	0.22	0.18	0.25	0.17	0.10	0.00	0.01	0.01	0.02	0.06	0.05
28	Phytone	Fat	/	0.02	0.06	0.04	0.00	0.04	0.05	0.01	0.02	0.04	/	/	/	/	/	/	/	/	/
**Alkenes**																					
29	β-Ocimene	Citrus, berbaceous, sweet	34	0.22	0.02	0.21	0.03	0.04	0.04	0.15	0.05	0.17	0.13	0.01	0.12	0.00	0.01	0.01	0.02	0.01	0.02
30	Naphthalene	Tar	/	0.08	0.05	0.05	0.01	0.01	0.01	0.03	0.02	0.03	/	/	/	/	/	/	/	/	/
31	Theaspirane	Camphor	/	0.10	0.03	0.01	0.06	0.03	0.01	0.08	0.03	0.02	/	/	/	/	/	/	/	/	/
32	*cis*-β-Farnesene	Floral, citrus	87	0.02	0.23	0.22	0.00	0.03	0.00	0.01	0.06	0.01	0.00	0.05	0.05	0.00	0.00	0.00	0.00	0.00	0.00
33	α-Muurolene	Wood	/	0.00	0.00	0.01	0.00	0.00	0.01	0.00	0.00	0.01	/	/	/	/	/	/	/	/	/
34	α-Calacorene	Wood	/	0.01	0.00	0.03	0.01	0.01	0.03	0.01	0.00	0.03	/	/	/	/	/	/	/	/	/
**Heterocyclic compounds**																					
35	Indole	Floral, animal-like	40	0.02	0.03	1.56	0.01	0.01	0.88	0.01	0.01	1.05	0.01	0.01	0.78	0.00	0.00	0.11	0.00	0.00	0.13
**Esters**																					
36	(*Z*)-3-hexenyl acetate	Green, banana	31	0.09	0.46	2.55	0.01	0.03	0.03	0.09	0.11	0.83	0.05	0.29	1.64	0.00	0.01	0.01	0.02	0.02	0.13
37	5-Oxotetrahydrofuran-2-carboxylic acid, ethyl ester	Roast, smoke	/	0.18	0.06	0.01	0.09	0.05	0.04	0.08	0.04	0.01	/	/	/	/	/	/	/	/	/

38	Methyl benzoate	Prune, lettuce, herb, sweet	/	0.27	0.06	8.95	0.01	0.00	0.77	0.06	0.04	7.89	/	/	/	/	/	/	/	/	/
39	Benzyl acetate	Fresh, boiled vegetable	/	2.63	0.54	35.56	0.06	0.04	7.26	0.54	0.33	23.81	/	/	/	/	/	/	/	/	/
40	Ethyl benzoate	Camomile, flower, celery, fruit	/	0.04	0.01	0.83	0.00	0.00	0.28	0.01	0.00	1.19	/	/	/	/	/	/	/	/	/
41	Methyl salicylate	Fresh, faint gingery, grass and milky	40	2.89	0.33	5.57	0.05	0.02	0.76	3.51	0.64	5.22	1.45	0.17	2.78	0.01	0.00	0.09	0.44	0.08	0.65
42	Ethyl phenylacetate	Fruit, sweet	/	0.02	0.01	0.01	0.00	0.00	0.00	0.21	0.05	0.04	/	/	/	/	/	/	/	/	/
43	Acetic acid, 2-phenylethyl ester	Rose, honey, tobacco	/	0.06	0.01	0.05	0.01	0.00	0.01	0.08	0.01	0.05	/	/	/	/	/	/	/	/	/
44	Ethyl salicylate	Wintergreen, mint	/	0.03	0.00	0.05	0.01	0.00	0.01	0.02	0.00	0.03	/	/	/	/	/	/	/	/	/
45	Benzyl butanoate	Plum	/	0.01	0.00	0.07	0.00	0.00	0.00	0.00	0.00	0.01	/	/	/	/	/	/	/	/	/
46	(Z)-3-Hexenyl hexanoate	Fruit, prune	/	0.22	0.00	0.01	0.00	0.01	0.00	0.01	0.00	0.01	/	/	/	/	/	/	/	/	/
47	Benzyl benzoate	Dairy	341	0.00	0.00	0.06	0.00	0.00	0.02	0.01	0.00	0.03	0.00	0.00	0.00	0.00	0.00	0.00	0.00	0.00	0.00
48	Isopropyl myristate	Fruit, rose, apple	/	0.00	0.00	0.00	0.00	0.00	0.00	0.00	0.00	0.00	/	/	/	/	/	/	/	/	/
49	Geranyl isovalerate	Odor description[a]	/	0.01	0.01	0.01	0.00	0.00	0.01	0.01	0.00	0.00	/	/	/	/	/	/	/	/	/

[*] Tea samples were instant teas prepared from three tea varieties, namely black (BT), green (GT) and jasmine tea (JT), using spray-drying (SD) and freeze-drying (FD).

[a, b] Odor descriptions, types and thresholds were found in the database (http://www.flavornet.org/) and literatures (18, 31).

[¶] rOAVs of raw tea materials were converted based on a tea to water ratio of 1:50 for brewing.

[§] rOAVs of the instant teas were converted based on a tea to water ratio of 1:200 for brewing.
